# Thirty-two Unselected Abdominal Sections with Tables

**Published:** 1891-12

**Authors:** Thomas Opie

**Affiliations:** Baltimore, Md.; Professor of Gynecology College of Physicians and Surgeons of Baltimore, and Gynecologist to the Baltimore City Hospital


					﻿11	.
Vol. viii.	DECEMBER, 1891.	No. 10.
<S)ricjina( (Somniiinicatione-.
THIRTY-TWO UNSELECTED ABDOMINAL SEC-
TIONS WITH TABLES.*
By THOMAS OP1E, M. D.,
lh-ofessor of Gynecology College of Physicians and Surgeons of Baltimore,
and Gynecologist to the Baltimore City Hospital.
Gentlemen—It is with no little diffidence and apprehension
that I offer this Society a paper which is the beaten track and the
hackneyed theme of a narration of cases, especially since it has
been made up little by little, as the fragments could be wrested
from the busy activities of the practical doctor.
I do not come before you with the promise to present new,
therefore interesting, facts. All of us might admit the paucity
of facts, even in the great province of medicine, yet all are ear-
nestly looking and longing for them. Every statement, propo-
*Read before the Southern Surgical and Gynecological Association, November, 1891.
sition or theory in any department of medicine, before it can be
reckoned among settled facts, must be submitted to a calm judi-
cial consideration, publicly and openly, before the bar of the med-
ical profession.
In new fields the caution and deliberation of the profession is
especially commendable. The experience of many observers is
desired; full and free discussion is sought; extensive statistics are
essential. In submitting this statement of a year’s work in ab-
dominal surgery, though it be small as compared with that of
many operators, it is to be hoped that it will elicit a full and frank
interchange of experience, aud thereby help in some measure
progress in this department of gynecological surgery.
In doing so I do not hesitate to say that every narrator of his
personal experience should, when before the tribunal of the pro-
fession, feel that the withholding of a single error of omission or
commission vitiates his confession. We want the truth, but more,
we earnestly desire the whole truth.
Statistics framed in this spirit would be invaluable to the pro-
fession, and, therefore; to the human family. In other words
“ every man should be his own critic.”
The accompanying tabulated statement embraces thirty-two
abdominal sections made in the twelve months, beginning No-
vember i, 1890, ending October 31, 1891.
This list does not include the whole number of laparotomies
performed at the Baltimore City Hospital during the time stated,
since this institution opens its doors to all operators of good pro-
fessional standing who may see fit to attend and operate on such
cases within its walls.
The writer had the misfortune to receive an attack of septic
empoisonment on December 10, 1889, from the prick of a pedicle
needle, while performing a laparotomy on a case of septic peri-
tonitis. This report therefor represents his first year’s work im-
mediately following that interruption, which lasted over a year.
It has been said, in connection with this important and rela-
tively new work, that there is “too much surgery.” In answer
to this charge I would say that this question can best be decided
by surgeons.
9
“ Everything is, by calm behest,
Resigned to him who un lerstands it best,
But every wordy, theoretic leech
Can teach the teacher how he ought to teach.”
It is a sad state of things if we can say of any individual sur-
geon that he is not wiser at the end than at the beginning of his
year’s work. He can, however, no more than the physican,
claim immunity from mistakes. If he be wise or skillful he will
not be chargeable with repetitions of the same mistakes; thus he,
as well as his future patients, will profit by his failures. Moreover,
there is inevitably an error in judging the work in this or any
other branch of medicine by the results of individual operators.
The whole of a thing can never be accurately determined by
any one of its parts.
The statistics in this new field are as yet comparatively small,
and the facts are few. It is quite well established that the hap-
penings incident to surgery will bear a very close relationship to
each other in every one hundred cases. It is equally true that
knowledge, calmness, deliberation, cleanliness and promptness
in coping with emergencies constitute the differences between
surgeons. A succinct definition for science is methodized
knowledge, but something must be added to cover the work of
the surgeon, viz., common sense and sound judgment. It is also
true that with a skillful surgeon each one hundred cases in his
own experience should be a success beyond its predecessor.
Would it not subserve a good purpose to adopt some uniformity
in our mode of collecting statistics ? It might be an improve-
ment if all members af this Society would alike report their work
in this department fully from one meeting to another, or at least
upon some uniform plan. As it is, the statements are frag-
mentary and, therefore, not so valuable, since successful cases
are given prominence and failures are apt to be overlooked. It
is agreeable to recount our successful cases; it is a very severe
test to confess our losses.
What there has been of error or detraction in my work I will,
as frankly and as fully as I know how to do, place before you
conspicuously in the early part of this statement, nor will I ask
you to “ hide the faults (you) I see.”
UNSELECTED CASES.
The operations were performed consecutively, as set forth in
the accompanying table. They were:
Ovarian tumors...........,.. .................... 6
Chronic ovaritis..................................7
Fibroid tumors....................................4
Pyosalpinx........................................5
Retroflexion with adhesionsand dysmenorrhoea. . . .3
Exploratory incisions.............................3
Extra-uterine pregnancy...........................1
Abscess of ovary..............................  .	1
Cyst of broad ligament........................  .	. 1
Cystic degeneration of ovary......................1
Total........................................32
Nine of these patients came to me through the dispensary
connected with the college and were operated on in the amphi-
theatre before the whole class at the College of Physicians and
Surgeons ; the remainder, twenty-three, were operated on pri-
vately. Twenty-seven were white and five were colored.
DEATHS.
The deaths were as follows :
Oophorectomy for pyosalpinx...................1
Shock from ovariotomy.........................1
Oophorectomy for acute mania..................1
Abdominal hysterectomy for fibro-cystic tumor.... 1
Total.................................. 4
No attempt will be made to describe fully each individual case
but the classification will admit of a separate discussion of the
various classes of disease or operations and enable the writer to
select and lay before the Association what seems of special
interest.
In such a report as this, the deaths are commonly regarded as
stigma; nevertheless they are usually instructive. I must give a
full account of my stewardship, and consequently trespass upon
your time, with the details of each one of them.
No. 7, colored servant, single, had received dispensary treat-
ment for several months for gonorrhoea. The diagnosis was
made out correctly, as the sequel proved, as gonorrhoeal pyosal-
pinx. The urgent necessity for surgical interference was im-
pressed upon the patient, but the operation was long deferred.
On the 5th of February,’91, a laparotomy was performed. Both
ovaries and both tubes were matted together and adherent to the
intestines in the posterior cul-de-sac. They were with difficulty
removed. The left tube burst in detaching it, and its virulent
contents were discharged into the peritoneal cavity. The pus
was easily and freely squeezed out of both tubes, after removal.
The abdomen was flushed out. Alarming hemorrhage ensued
from the denuded surfaces of the cul-de sac which was arrested
by packing it firmly with iodoform gauze. This was removed
in six hours. Her temperature rose to 101 degrees during the
night, and reached 103 degrees the' next day; pulse 130. She
died on the third day of septic peritonitis.
Post Mortem: Flaky lymph and pus over intestines. No
fluid in abdomen. Ligatures were holding securely.
Case io.—W., age 50, married; visited me at the hospital
about February, ’91 received an opinion that she had an ovarian
tumor which was fully developed and should be removed at once.
This advice was disregarded. She returned home and remained
there until about six or seven weeks later, when a letter to her
physician impelled her to come on for the much needed surgical
treatment. She was admitted on the 16th of March and oper-
ated on on the 19th. Her condition was bad. Her temperature
was 100, and pulse rapid. Her abdomen was very sore under
palpation. The colloid contents and the solid elements of the
tumor weighed over twenty pounds. The whole abdominal
wall seemed ablaze with inflammation; the intestines were
adherent largely to the tumor and matted together. The con-
tents of tumor would not run, but had to be scraped out piece-
meal by the hand. She was profoundly shocked ; hypoderms of
whiskey were given freely; milk, beef tea and whiskey were
administered per rectum ; though retained, they were of no avail.
Death ensued at 3 a. m., I think from surgical and chloroform
shock.
Post mortem by Prof. Kierle: No blood in abdomen whatever ;
abdominal cavity perfectly dry. Ligatures tightly adherent to
parts. Organs healthy. Died of shock.
Case 18.—W., age 18, single, family history good. Had had
periodical attacks of mania. The first attack came on simultane-
ously with her menstrual flow at 15 years of age. Her men-
struation was always accompanied by severe pain. Six months
ago she had so severe an attack of mania, that she was forced
to go to an insane asylum. On the third day after the opera-
tion mania set in ; she could not be kept in bed. It was impos-
sible to keep her bandage on. She grew more and more violent
and died January 23, 1891—thirteen days after the operation.
Post mortem by Prof. Kierle: Complete post mortem not
allowed. Kidneys markedly congested. No fluid in abdomen.
Lungs congested. Brain not examined ; probable cause of death
here.
No. 32.—W. M., white, married when 21 years of age ; six-
teen years ago ; had three children and one miscarriage.
Five years ago noticed tumor in her abdomen, which gradu-
ally grew larger and was said to be an ovarian cystoma. She
suffered from hemorrhages very often. They were profuse in
character and at irregular intervals. On examination a tumor
as large as a man’s bead was realized in the abdomen. The ute-
rine probe ran up into it thr.ee-fourths of its length. An abdominal
incision was made about eight inches long ; the tumor was lifted
out of abdomen and secured by Bieker Brown’s clamp. The
bleeding was readily controlled and the peritoneal cavity flushed.
The abdominal wall was closed with silk-worm gut and the stump
secured at the lower angle of the wound. Though severely
shocked she rallied well. The mass comprising the fibroid,
womb, ovaries and tubes weighed ten pounds.
The patient did well for three or four days, but after this her
pulse and temperature began to rise, and she died on the seventh
day of septic peritonitis.
Post mortem, by Prof. N. Gr. Kierle: Wound had entirely
healed up ; the clamp was tightly holding the stump. Abdomen
contained two ounces of bloody fluid. The intestines were ad-
herent and covered with inflammatory lymph. Kidneys soft and
fatty ; liver fatty. Death from septic peritonitis.
EXPLORATORY LAPAROTOMY.
No. 4.—Age 46; operated on January 5th, 1891. The explo-
ration revealed malignant degeneration of the left ovary, with
cancerous cysts studding the peritoneum and intestines. A large
amount of abdominal fluid was removed. The cavity was
thoroughly flushed and the walls closed. Patient returned home
in three weeks greatly improved. It must needs have been,
temporary.
No. 22.— Age 25, married, sterile. Operated August 26th,
1891. A large malignant growth was found in the left side involv-
ing the corresponding ovary and the liver. Patient had icterus
at the time of operation Stitches removed on the eighth day.
She improved rapidly and returned to her home in good spirits.
M. O., operation, September 26th, 1891; age 33, widow; had
two children and one miscarriage during wedlock. Performed
criminal abortion with a tortoise shell bonnet pin. On admission
a digital examination was made. The pelvis was blocked with
exudates. In the center of the post cul-de-sac was a boggy
point, the field all around solid to the touch. Percussion and
palpation indicated the extension of the inflammation and effusion
high up on the left side of the abdomen. The temperature in
the mornings was about 10 £ degrees; in the evenings about 102 y2
to 103 degrees.
An exploratory incision was decided upon, and made in the
median line. It was impossible to explore the pelvis; the strong
resistant adhesions and exudation was an effectual stay law. A
large amount of bloody serum was flashed out. The tempera -
ture at the time of operation, 102 degrees, began to decline at
once; the effusion was gradually absorbed and at the end of
three weeks there was no sign of the pelvic trouble. Drainage
was not used in any one of the three cases.
The number of these cases is too small to prove anything, but
they are suggestive and add to already strong testimony which
is recorded, that a laparotomy done in thoroughly aseptic manner
is a warrantable resort, when the indications are threatening and
the diagnosis doubtful.
OVARIAN TUMORS.
The six cases of ovariotomy made good recoveries with the
exception of No. io, in which there was realized extensive
peritonitis on opening the abdomen. This case is reported
among the deaths.
No. 2.—W., single, age 38, was an interesting case, since in
addition to the cystoma of the right ovary, which contained two
and a half gallons of fluid, there was on the left side a dermoid
cyst the size of a child’s head. The contents consisted of bone,
hair, etc. The pedicle of this tumor was four inches wide, hence
there was some difficulty in constricting it efficiently, even in sec-
tions. This patient, strange to say, menstruated regularly up to
August 25th last, within three months of the time of operation.
She suffered from attacks of mental aberration during a year prior
to the operation. Excellent recovery ensued both mentally and
physically, which has been maintained without interruption from
the time of the operation up to this date.
No. 21.—W., single, age 16. Her abdomen approximated
the size of a woman’s at full term, before the operation. She and
her friends noticed the enlargement of her abdomen for the first
time four years ago, when she was twelve years of age. It is
fair to conjecture that it had filled the pelvis and like the preg-
nant uterus had developed into the abdominal cavity at that time.
If there is a parallel between the nutritive growths in these two
conditions, this tumor must have started at a very early age,
possibly when she was eight or ten years old. Her first menstrua-
tion was in January last, and it recurred irregularly afterwards.
She bore the operation and the subsequent treatment with the
greatest fortitude and made an excellent recovery.
The other four cases of ovarian tumor presented nothing of
special interest, save their complete and permanent recovery.
CHRONIC OVARITIS.
In this class there were six. Among them was case No. 17,
single, age 20. She began to menstruate at 15. Her first epi-
leptiform spasm began coincidently with this event. From that
time up to the time of the operation, she had these attacks both
during the interim and at the time of the menses. The attacks
were more severe just preceding the establishment of the flow.
They were attended by convulsive movements of a pronounced
character, followed by a period of stupor or sleep lasting fifteen
or twenty minutes. At other times they were caused by over-
tax, by mental worry or excitement. The intermenstrual spasms
were attended by slight twitchings and a short sleep. Her
friends, prior to seeking relief by oophorectomy, had spent quite
a fortune in their efforts to cure her, having had her under the
treatment of some of the most eminent neurologists in New
York. Electricity, massage, drugs, all in turn proved unavailing.
No difficulties arose in the operation. A few days after the op-
eration she had several spasms, but no harm ensued from them.
Convalescence was uninterrupted and rapid. She spent several
weeks at the seashore and paid me a visit on her return home.
The young lady had gained notably in weight and strength, but
the intermenstrual form of the attacks still recurred. At this
writing I am unable to state her condition.
Case 18.—White, single, 18 years of age, was an interesting
case of this class, who had an attack of acute mania at the first
menstrua] flow. Six months ago she had a recurrence of insanity,
and was sent to an insane asylum. Oophorectomy was followed
by acute mania. She was uncontrollable. Death ensued from
sepsis. The case has already been reported in this paper under
the caption of the deaths.
Case 12.—White, single, age 21. Had always been sick since
childhood ; had been for years subject to recurrent attacks of
follicular tonsilitis. Her menses were established at 17 ; about
that time she had a severe attack of typhoid fever. For six
months prior to the operation of oophorectomy she had been
bedridden. She was hopeless, dyspeptic and anaemic in the ex-
treme degree. Her neuralgic headaches and ovarian pains were
intolerable at each menstrual epoch. She readily accepted the
proposal as to removal of the ovaries. The operation was borne
courageously, and her convalescence was uninterruptedly good.
Upon her return to her home in Baltimore she relapsed into her
former despondent condition. She has not fulfilled my expecta-
tions as to complete cure, though she has improved physically.
No. 14.—Age 23, white, single. Was healthy until 13, when
menstruation began. At first the recurrences of it were painless
and regular. An interruption of four months occurred and dys-
menorrhoea, menorrhagia and ill health followed. Tormented
by her physical pains and disqualification and her inability to
support her aged parents, she sought oophorectomy as a last re-
sort. It has brought about excellent health and capabilities.
No. 16.—White, aged 22.	S., unlike the preceding case, had
led a life of luxury and ease. Her dysmenorrhoeal pains in def-
ecation and general depreciation in health during five years caused
her family to seek the removal of the ovaries. Perfect satisfac-
tion as to health, cheerfulness and comfort has come both to her
and her friends.
No. 27.—Age 39, operation October Sth, colored, a washer-
woman, widow. Had one full term child and one miscarriage.
This patient was operated on by me one year ago for a deep
laceration of the cervix. While the parts healed well, she was
not benefited as regards her distressing and disqualifying dys-
menorrhcea. The appendages were removed. She is still in the
hospital. All the indications betoken a happy issue out of her
afflictions.
It is noteworthy that five out of six of these cases of chronic
ovaritis were single, and that their ages range from 18 to 23
years.
PYOSALPINX.
The five cases under this heading all made good recoveries
was a very unpromising one. Both ovaries and
tubes were packed in one conglomerate mass in the posterior
cul-de-sac; one of th<* t,ubes ruptured on removal, causing septi-
CcEmia. It is interesting to relate, as bearing on the etiology of
this case, that every one of these cases was undoubtedly gon-
orrhoeal in origin.
Two had been under treatment in our dispensary by Dr. W.
S. Gardner, and the other three were ladies who were innocent
victims to the viciousness of their husbands. I prescribed for
one of the males a short time prior to marriage for gonorrhoea,
and was greatly astonished, not long after that event, to find him
with a bride whom he had infected by the same disease. Six
months later the uterine appendages were removed. The other
two confessed to having transmitted it to their wives. One of the
cases was an extremely critical one, since a pus tube had burst
and discharged through the rectum three months prior to the op-
eration. From that date to the operation the lady was disquali-
fied for any household duty, though she had around her a large
family. When operated on, six months ago, she weighed 130
pounds; she now weighs 160 pounds, her weight when in former
good health.
FIBROID TUMORS.
Two abdominal hysterectomies were performed for fibro-cystic
degeneration of the uterus. The first was :
No. 11.—The abdominal incision was fourteen and a half
inches long from the sternum to the pubis. The tumor weighed
twenty pounds. Its vertical circumference measured twenty-three
and a half inches, and its transverse circumference twenty-two
and three-fourths. There was, in view of the very large pedicle,
considerable difficulty in securing her against hemorrhage; hence
the intra-abdominal method of treatment would most likely have
failed had it been done. A full description of this case has al-
ready been published in the proceedings of the A. M. A. of 1891.
The patient was unburdened and health is now being enjoyed.
The second was a case of supra-vaginal hysterectomy for a
fibro-cystic tumor of the uterus. This has been described.
The third case of fibroids gave the following history: Her first
intimation of a tumor was October 18, 1890. While playing on
the piano she had a rush of blood which filled a chamber. This
was the time of her menstrual flow. She bled alarmingly at
each menstruation. Prior to operation she had bled continu-
ously for a month. Diagnosis was intramural fibroids, chiefly
occupying the posterior wall. The pelvis was well filled by the
tumor. While I hoped to find the ovaries, due preparation was
made for a hysterectomy. They were happily in front and were
removed. Not a drop of blood has appeared since the opera-
tion. She made a good recovery.
Case 5 was one of sub-peritoneal fibroids. The p itient had
been bedridden for six months, though sick and disqualified for
all the duties of a wife for years. The uterus was retroflexed by
a fibroma, the size of a hen’s egg, situated on the upper posterior
part of the fundus. In addition to this, there was a small in-
tramural fibroid, the size of a filbert, located on the posterior
wall, at the junction of the body and neck. Myomectomy was
done, and both ovaries and tubes were removed. She has made
a good recovery.
HYSTERRORRHAPHY.
In two cases, No. 1 and No. 25, hysterrorrhaphy was per-
formed, after the removal of the appendages, for retroflexion of
the uterus with chronic ovaritis and dysmenorrhoea.
The operation by suturing through the anterior uterine wall
may well be considered obsolete. The first case I performed on
13th November last, suturing through the stump of the uterine
appendages, without scraping or otherwise injuring the uterine
wall. The outer parts of the sutures were cut off at the end of
two weeks, and the remaining parts allowed to fall back into the
abdomen.
In the second case, No. 25, the sutures were simply made
to pierce (without tying) the ovarian ligament and brought
through the abdominal wall opposite their respective insertions
about one and one-half inches from the incision, and tied over a
bridge of skin one-half inch wide. In two weeks they were re-
moved by cutting one side and drawing them out entirely.
Both cases, when discharged, were in excellent condition. The
uterus, in each case, was well secured in its rectified position, as
attested by several competent examiners. Farther time is nec-
essary to establish the permanency and value of these operations.
CYSTIC DEGENERATION 9F OVARY, COMPLICATED BY PREGNANCY.
No. 23, M., 23 years old, has had no full term children, but
three miscarriages during the first third of gestation. Patient
had been subjected to considerable local treatment without avail.
Her present trouble with the left ovary had been recognized
during the past three years. The possibility of pregnancy was
broached, but her attending physician said he had lately been
making intra-uterine applications, and hence was confident she
could not be pregnant.
On opening the abdomen, the diseased ovary was readily ver-
ified, but the uterus was unusually large and the cervix was
dilated with parallel bar dilator, so as to explore its contents. A
foetus of six weeks was withdrawn ; the ovaries were removed.
The subsequent history of the case was uneventful save that re-
covery was rapid and complete. The ovary on left side was
represented in the thickened portion of the wall of a cyst the
size of a walnut.
EXTRA-UTERINE PREGNANCY IN A DOUBLE UTERUS. REMOVAL
OF SAC AND THE REMAINING OVARY.
No. 30, age 32, had been married fifteen years, but was with-
out offspring. Since girlhood she had been regular with men-
struation. For four months past her menstruation had been
scanty and irregular and her general health miserable. One
month prior to the operation she was compelled to go to bed.
When the patient was first examined, in consultation with Dr.
Wm. Gombel, of Baltimore, her attending physician, there was
a hard resistant mass occupying the left side of the pelvis, press-
ing upon the rectum and causing intolerable agony. Laparot-
omy was determined upon. A large encysted mass, the size of
two fists, occupied the posterior and lateral regions of the pelvic
basin, pushing the double uterus to the opposite side. At the
outset, in enucleating it, the blood contents escaped, the sac was
shelled out and the pedicle ligatured. The right ovary was re-
moved. The abdomen was flashed, the drainage tube inserted
and the abdomen closed.
The double uterus was realized by sight as well as touch, it
having been held up in the incision for a satisfactory recognition
by all present. There were two distinct funduses with a deep
sulcus between them. The cervix was single. The drainage
tube was taken out in twenty-four hours. The patient, during
about ten days, although doing well physically, had an attack of
mania. Providentially, she was at no time unmanageable, though
she was watched with the most assiduous attention by her nurse.
She entertained two vagaries, but aside from them, was sane and
logical. One was the constant dread that she was to be oper-
ated on by the doctor again, and when either of her attendants
entered the room she was frantic with fright. The other de-
parture was that she could hear her family talking in the next
room. She would answer their supposed questions and plead
pitifully that they be admitted. She is now perfectly sane, has
gotten well of her stitch abscess, which followed the drain tube
and is out of harm’s way. An examination of the tissue of the
sac showed it to be placental.
STITCH ABSCESS.
This complication occurred nine times, a much larger number
relatively than I have seen recorded heretofore; while no case
proved disastrous, several were exceedingly annoying in delay-
ing patients in hospital. They occur most frequently in cases
where the drain tube has been used. The early opening of ab-
dominal dressings, for any purpose, favors their occurrence.
When the dressings remained intact for seven days there seemed
to be greatest immunity from the stitch abscess. Dr. Welch
says, “ A coccus, which may appropriately be called the staphy-
lococcus epidermis albus, is a nearly, if not quite constant inhab-
itant of the epidermis, lying both superficially and also deeper
than can be reached by present methods of disinfection of the
skin. The coccus is found frequently in aseptic wounds. It may
be the cause of disturbances, usually of a relatively slight de-
gree, in the healing of the wound, especially when drainage
tubes are inserted. It is the most common cause of stitch ab-
scesses in wounds treated aseptically and anti-septically.”
DRAINAGE IN ABDOMINAL SURGERY.
Drainage was resorted to in but three cases during the year.
Case 2, ovarian and dermoid cyst, had a drainage tube in
five or six days, and I am convinced it retarded her convalescence.
No. 28, extra-uterine pregnancy, had a tube in less than twen-
ty-four hours. If I may judge of the necessity for it by the
quantity or quality of the discharge through it, I should say it
did no good. A small superficial abscess at the entrance of the
tube followed its withdrawal.
No. 32, ovarian abscess, had a drain tube in about twenty-
four hours. An abscess occurred at the site of its entrance. The
quart of pus, which was saculated in this case, was removed with-
out an atomic part of it touching the peritoneum or the wounded
parts, otherwise her fate would have been sealed, as was the case
in No. 7, where the pus tube burst and death ensued on the third
day of peritonitis. Even in such a case as the latter, the most
we could do would be to thoroughly flush out the abdomen.
I am of the opinion that there is even too much flushing done;
it is but seldom called for. A plentiful supply of fine, properly
prepared elephant ear sponges will do away with flushings in
most cases and remove the necessity for drainage. They are
efficient helps in keeping the abdomen free from infection. They
can be utilized in keeping back the intestines, in occupying the
cul-de-sac, in positions below the pedicle, in taking up blood or
secretions, in staunching hemorrhage, in separating adhesions,
in protecting the intestines while closing the abdomen. The as-
siduous personal attention of certain workers using the drain tubes
has caused them to escape the disasters which have befallen
the less careful and less skillful surgeons.
Nature’s plan for curing the unsightly rents the surgeon makes
when he opens the abdominal cavity is to seal hermetically the
sacred cavity of the peritoneum in twenty-four hours. This
kindly and providentially comports with its sensitiveness and its
fitness for the cultivation of germs of disease. Does not this
prompt sealing of the peritoneum speak with unmistakable
logic to the point of striving hard for an aseptic operation and for
securing immediate and absolute closure ?
The oft-repeated removal of the dressing of the patulous
drainage tube must of necessity be a very great danger ; surely
it favors decomposition and invites germs. All surgeons are
aware that after an anaesthetic, restlessness and jactitations are
not wholly restrainable. It is easy to conceive how physical in-
jury may accrue to the patient during this critical time from
hese not at all innocent, yet smooth, glass tubes.
I believe drain.ige is doing more harm than good and, there-
fore, ought to be abandoned by the abdominal surgeon.
There is a dual personality as well as power concerned in all
surgical work. The one is the surgeon, who skillfully meets and
disposes of the crisis in the more mechanical part of the work
and, therefore, receives the plaudits of the multitude ; the other
is the influence behind the throne, more potent than the throne
itself, which reaches beyond the eye, the touch and the knife. I
scarcely need say it is the modest, yet oft-despised, laboratory
physician who is teaching us the hidden leaven of disease. Let
us give him grateful recognition, for the pivotal facts and secret
springs in recent surgical success. When he says bruised tissue
is a paragon field for the cultivation of infectious germs, let us
heed the warning and cast aside the drainage tube.
Dr. Park says as to drainage: “ Views and practices concern-
ing drainage have materially changed even since the antiseptic
era began. Our predecessors drained to permit escape of pus,
which they knew would form. Until lately we have drained
in order to prevent its formation. We seem now to be on the
eve of an era when we need to drain but little or not at all. We
resort to drainage now only of necessity, in septic or infected
cases. In other cases we drain mainly from habit, or from fear.
Indeed, when we start afresh, as it were, without previous infec-
tion, the practice of drainage is a confession of fear or of weak-
ness, both of which are alike unscientific and unfortunate. It
even seems to me that in many cases where all other aseptic
requirements have been met, we do much more harm than good
by the use of drains.”
TABLE OF ABDOMINAL SECTIONS AT BALTI-
MORE CITY HOSPITAL,
By THOMAS OPIE, M. D.
ABDOMINAL SECTIONS.
NColor.d Name- AKe- Date- Single. Married.	Disease.
1
H. T. 30 Nov. 13, ............ M.	5	.......Retroflexion dysmen-
W.	1890.	orrhoea.
|	i
2	i
{ W. E. 38 Nov. 24, S................................... Ovarian tumor and
W. I	1890.	dermoid cyst.
_____J	______ 
3	Chronic ovaritis dys-
H. L. 37 Nov. 28, S.................................. menorrhcea and hys-
«W.	1890.	teria.
____________________________________________________________________________________
1	Jan. 4,
B. C. 46	1891.......... M. .....................Cancer of the ovary.
W.
5	Jan. 9,
P. N. 47	1891.......... M. .....................Subperitoneal fibroids.
W.
I
6	j	Jan. 28,
| R. S. 32	1891.	....... M.	5	.......Gonorrhoeal pyosal-
W.	|	I	pinx.
_________I ______________________I_____________________I_______________________________
7	Feb. 2,	Gonorrhoeal double
S. J. 21	1891. S................................ pyosalpinx. Exten-
B.	sive adhesions.
8	Feb. 9,	Gonorrhoeal salpingi-
S. A. 22	1891.......... M. ..................... tis, chronic ovari-
W.	tis.
9	Feb. 22,
S. B. 28	1891..........Widow....................I Ovarian tumor,
wy
10	March 19,	j	Ovarian tumor. Peri-
W. S. 50	1891.......... M..............|........ tonitis.
W.
BALTIMORE CITY HOSPITAL.
Operation.	I I)rain’ ^tions^' Recd- Died.	Remarks.
Examination before leaving the
Removal of both ova-	Stitch ab-	hospital showed the displace,
ries. Hysterorrhaphy, j No. scess.	R........... ment to have been corrected.
Health restored.
Right ovary distended with 2*^
gallons fluid. Nearly its entire
surface was adherent to ab lom-
Extirpation of both tu-	inal wall. On the left side was
mors.	Yes............. R.............. a dermoid cyst the size of a
child’s head, the pedicle of
which was four inches wide.
Recovery complete.
A working woman totally disa-
bled from making a living ; was
Removal of both ova-	]	anaemic, subj set to dysmenor-
ries.	No.............-.	R. ...... rhoeal pains, vertigo and faint-
ing tits. Health has been en-
tirely restored.
Large cancerous cysts studded the
peritoneum and intestines, ova-
ries had undergone malignant
Exploratory laparoto-	degeneration. Abdominal fluid
my.	No.............. R.............. was removed, the cavity flushed.
Patient returned home in three
weeks much improved.
Had been bedridden for 6 months,
though sick for years. Uterus
was retroflexed by a fibroid the
Myomectomy, oophorec-	size of a hen’s egg, on the upper
tomy.	No.............. R...........1	posterior aspect of the fundus.
j This was removed. A small
1 fibroid located on posterior wall
1 of uterus. Hence oophorecto-
i my. Result permanently good.
[On the fourth day after operation
Removal of both ovaries	| menstruation seemed to be at
and tubes.	No.............. R...........'	hand and lasted four days.
;	I Made an excellent recovery and
| has seen no show since.
Patient died of sepsis on the third
A pus tube	day. The whole of Douglas’
Removal of both ovaries	burst; hem	. cul-de-sac was denuded and
and tubes.	Yes. orrhage .............. D.	from this surface copio is hem-
was very	orrhage ensued Bleeding was
great/	arrested by packing with iodo-
form gauze.
[Anaemia most pronounced; com-
plexion waxy; general health
Removal of both ovaries	very bad, so much so as to ren-
and tubes.	No................. R........... der doubtful the advisability
of operation. The gravity of
the case stated to the friends.
.	The results most satisfactory.
Patient noticed her abdomen was
growing larger when she was
Removal of tumor and	twelve. She had her menses
the other ovary.	No. Stitch ab- R................ last winter several times, but
scess.	the flow was scanty. Her periods
were irregular. Had an unin-
terrupted and rapid recovery.
[Found a large cancerous mass
Exploratory laparoto-	j in the left side involving the
my.	No.............. R. ......] liver as well as ovary. Closed
the abdomen. Recovered suflfl-
;	| ciently to go home.
ABDOMINAL SECTIONS.
NColor.d Name- I Age. Date. Single. Married. I	^ried.”'	Disease.
______f__________I_____________________________I______2___ ’______________________
11	April 20,
T. V. L. 48	1891............ M.	...............Fibro-cystic tumor of
W.	I	uterus.
12	April 21,
II. J. 21	1891. S......................!....... Chronic ovaritis, dys-
W.	I	rnenorrhcea.
I
I -	!L !
Uterine retroflexion.
Abscess involving
13	April 26,	right tube and ovary
S. M. 35	1891............ M.	5	....... discharged through
W.	rectum. Painful
mass on right side of
pelvis.
I
14	May 9,
A. B. 23 I 1891. S..................|.......j.......Dysmenorrhoea, anae-
W. I	i	mia, metrorrhagia.
(Ovarian tumor of right
15	i j May 11,	j	i ovary,left ovary had
S. E. 28 ;	1891.	......I M.	3	........j a cyst in it contain-
W.	I	I ing 10 ounces fluid.
16	May 20,	Chronic ovaritis, dys-
W. A. 22	1891. S. ............................ menorrhoea, anae-
W.	mia.
17	June 3,
S. K. 20	1891. S.................. ...........Epileptiform spasms,
W.	dysmenorrhoea.
18	June 18,	Acute mania, dysmen-
V. M. 18	1891. S.............................. orrhoea.
VV.
19	July 4,	I	Traumatic peritoni-
S. M.L. 25	1891............ M.	.......'....... tis, retroflexion, dis-
W.	I	placed and diseased
ovary. •
BALTIMORE CITY HOSPITAL.
Operation.	Drain. C°y^sCa' Rec’d. Died.	Remarks.
Examination disclosed enlarged
and cystic ovaries. Her physi-
cian had probed the uterus and
Removal of both ovaries	was satisfied she could not be
and a foetus of six	pregnant. She had had three
weeks.	No. Pregnancy R................. miscarriages in the first-third of
.	"	gestation. Determined after
conference to remove the foetus
and continue operation. Result
excellent.
Had expected to do a hysterecto-
my, as uterus was large and it
was unlikely that the ovaries
Removal of the ovaries.) No. Stitch ab-	could be reached, but both were
scess.	R.......... in front and easily removed.
Patient had some metroperi-
tonitis for several days, but
made an excellent recovery.
Hemorrhage readily controlled.
Blood removed thoroughly by
sponges, which had been packed
Removal of both ova-'	fin cul-de-sac. No drainage, no
ries, including the ceJ-	flushing. Temperature did not
lulitic mass involving,	j	i	rise above 100°. Stitches re-
the right ovary.	) No. 1.............. R............ moved on tenth day. Recovery
rapid. In five months has in-
creased in weight from 130 lbs.,
at time of operation, to 160 lbs.
! Health excellent.
,	A factory hand who had been
Removal of both ovaries	,	| compelled to quit work on ac-
and tubes.	No................ R............ count of ill health, has been
j able to resume work, is in good
health and spirits.
Removal of tumor and No. Stitch ab-	| [Made an excellent recovery ex-
the other.	scess.	R. I......| cept as to the delay from the
| stitch abscess.
Elevation of temperature com-
i	bated successfully by salines.
I Two stitches removed on fifth
Removal of both ovaries	•	day and the others on the sev
and tubes.	No. Stitch ab-	enth day. because of redness
scess. R............... around them. Superficial sup-
puration continued two weeks ;
healing rapid. Thorough re-
i	covery.
Menstruation begun at fifteen,
since then had been at every
recurrence subject to fits. A
number of distinguished nerve
Removal of both ovaries	specialists in New York and
and tubes.	No................ R............ elsewhere had treated her.
Massage, electricity, drugs, all
, alike failed. Recovery from op-
eration excellent. Cure incom-
plete.
Had periodical attacks of mania
with menses since first appear-
ance at fifteen. Six months
Removal of both ovaries	ago was in an insane asylum,
and tubes.	No........................ ^D. A few days after operation,
acute mania set in. She died,
the eleventh day after opera-
tion.
Uterus retroflexed and bound by
adhesions in its malposition.
Removal of both ovaries	I Had cystitis. Was always in
and tubes.	No. Cystitis. R.................' pain. Menstruation painful and
irregular. Cause : A fall when
fourteen years old. Recovery
i	complete.
ABDOMINAL SECTIONS.
NColor.d Name- ASe- Date. Single. Married. ^ren.	'	Disease.
20	July 18,
D. K. 42	1891. S..............................Ovarian tumor.
B.
21	Juh 23,
W. A. S. 16	1891. S..................................Ovarian tumor.
W.
■	;L I 
•
22	Sept. 1,	Cancer of left ovary
L.	C. 25	1891........... M. ...................... involving the intes-
W.	tines.
I________________I__________________________________________•__________________
I
Both ovaries cystic.
23	Sept. 2, |	The leftmass of cysts
W. E. 23	1891.	...... M........................ displaced in poste-
W.	rior cul-de sac and
adherent.
21	Sept. 9,	Intramural fibroids.
R. R. 27	1891. S.................................... Dysmenorrhcea.men-
B.	orrhagia alarming in
extent.
25	Sept. 24,	Retroflexion, prolaps-
R. A. 33	1891........... M.	2	1 ed ovaries, dysmen-
W.	orrlmea.
 
26	I Sept. 26,	Peritonitis, haemato-
M.	O. 33 I 1891...................................... cele, abortion.
W.
27*	Oct. 8,	I	:	I
C. M. 39	1891........... Widow. 1	1	1 [Chronic ovaritis, dys
B.	I	II menorrhoea.
28
S. M. L. 25 | Oct. 18, ....... M........................Cyst in broad ligament
W.	I 1891.	*
I
BALTIMORE CITY HOSPITAL.
Operation.	Drain.	I Rec’d. Died.	Remarks.
Patient noticed the growth six
years ago, but did not know
what it was. It was claimed by
Removal of tumor and	her that menopause came on at
the other ovary.	No. Stitch ab-	thirty-six, and growth was
scess.	R......... more rapid the last three years.
When she left hospital the ab-
scess had not completely healed.
Recovery is complete.
aijo-ht	j IMade a complete recovery. Have
Removal of tumor and	stdch	!	1 seen her lately in good health,
other ovary.	No.	R. I......: Has resumed her wonted weight
______________________________aoscess.	I	j and vjgOr,_____________________
Patient called six weeks ^before
operation and was, in view of
the great abdominal develop-
ment, advised to ac 'ept imme-
Removal of tumor and	diate operation. When she ar-
other ovary.	No........................ D.	rived she was suffering with pe-
ritonitis Death took place
two hours after the operation,
from surgical and chloroform
shock:.
Incision 14% inches. Tumor
weighed 20 pounds, its vertical
circumference was 23% inches
Abdominal hysterecto-	! and its transverse 22% inches,
my.	No.............. R. ......' Treated stump extra-peritone-
ally. Temperature did not
reach beyond 9.9°, nor pulse be-
■ low 30. Recovery was uninter-
1 rupted and complete.
Had been bedridden for over six
months. Had been a sufferer
with dysmenorrhoea since sev-
enteen, when her menstruation
Removal of both ovaries No............... R. !......... begin. She gained in weight
and was cheerful for a while,
but relapsed into toe same neu-
rotic state as before operation.
Improved physically, not- men-
_________________________________________________________tally.________________________
Has suffered for years with retro-
flexion and prolapse of ovary ;
Oophorectomy. Hyster-	| was disabled for all duty. Re-
rorrhapliy.	No. Stitch ab- R..............I	moved both ovaries and stitched
scess.	the uterus to the abdominal
wall. Made a good recovery.
(Time of operation, temperature
102°. It began to fall at once.
Exploratory laparotomy No................ R............ in three weeks the exudates
I were removed. Has made a
| perfect recovery.
Nothing worthy of comment save
Oophorectomy.	No. ............ R............ relief from pain and promise of
complete recovery.
Same case was operited on July
4, 1891. Cyst of broad ligament
Laparotomy for removal	containing serum removed. En-
of cyst.	No.............. R............ tire relief from pain. Rec >very
uninterrupted. Is now sitting
up-
ABDOMINAL SECTIONS.
N°oord Name> ASe- Date- sinSle- Married, °gl.	Disease
29	Oct. 18,
G.L. 32	1891........... M................... 1	Extra-uterine preg-
W.	nancy.
30	I	Oct. 15,
B.L. 37	1891........... M.	4	1 Pyosalpinx.
W.
31.	Oct. 17,
B. M. 32	1891........... M................... 1	Ovarian abscess.
B.
32	|	Oct. 29,
i W. M. 37	1891........... M.	3	........Fibro-cystic tumor of
uterus.
BALTIMORE CITY HOSPITAL.
Operation.	Drain. ^'°tp)^Ca' Rec’d. Died.	Remarks.
Four months had scanty, irregu-
lar and painful menstruation.
She was compelled to go to bed
Laparotomy, removal of	one month ago. On opening ab-
sac, with placenta and	domen a large sac in left iliac
both ovaries.	I Yes. Mania of a	region strongly adherent to sur
mild type. R. .......... rounding structures, involving
tube and ovary. Microscopic
examination showed placental
tissue. Drain tube used twenty-
|	four hours. Recovered.
Had a clear history of gonor-
rhoea ; was under dispensary
Removal of both ovaries!	| treatment for three months,
and both tubes.	j No.............. R. ........... Flushing was used; no drainage.
Recovered without unfavorable
conditions.
Sac filled the pelvis nearly to the
umbilicus, was at every point
Extirpation of abscess	adherent to uterus, intestines or
sac and other ovary	Stitch ab-	abdominal wall—was enucleated
and tube.	‘ Yes. scess. R.................. by the hands rather than the
knife. Drainage tube used
twenty-four hours. Her recov-
ery is to be regarded as phe-
nomenal.
Tumor began to show five years
ago. Since then has been suf-
fering profuse hemorrhages.
Was supposed to be an ovarian
Abdominal hysterecto-	tumor. Got through operation
my.	No........................ D.	well. The stump secured by the
Baker-Brown clamp. No bleed-
ing. About the fifth day tem-
perature up to 103°; pulse 150.
Death took place on the seventh
I I	|day from peritonitis.
				

